# Mitophagy in atherosclerosis: from mechanism to therapy

**DOI:** 10.3389/fimmu.2023.1165507

**Published:** 2023-05-16

**Authors:** Yanhong Zhang, Jiajun Weng, Luyao Huan, Song Sheng, Fengqin Xu

**Affiliations:** ^1^ Xiyuan Hospital, China Academy of Chinese Medical Sciences, Beijing, China; ^2^ Traditional Chinese Medicine Clinical Medical School (Xiyuan), Peking University, Beijing, China; ^3^ Department of Integrated Traditional and Western Medicine, Peking University Health Science Center, Beijing, China; ^4^ Graduate School of Beijing University of Chinese Medicine, Beijing, China

**Keywords:** atherosclerosis, mitophagy, different cell types, mechanism progression, drug and natural product progression

## Abstract

Mitophagy is a type of autophagy that can selectively eliminate damaged and depolarized mitochondria to maintain mitochondrial activity and cellular homeostasis. Several pathways have been found to participate in different steps of mitophagy. Mitophagy plays a significant role in the homeostasis and physiological function of vascular endothelial cells, vascular smooth muscle cells, and macrophages, and is involved in the development of atherosclerosis (AS). At present, many medications and natural chemicals have been shown to alter mitophagy and slow the progression of AS. This review serves as an introduction to the field of mitophagy for researchers interested in targeting this pathway as part of a potential AS management strategy.

## 1 Introduction

Atherosclerosis (AS) is a systemic disease and an important risk factor for various high-risk cardiovascular and cerebrovascular diseases, such as stroke, myocardial infarction, and even sudden death (1). Vascular endothelial cells (ECs), vascular smooth muscle cells (VSMCs), and macrophages all play important roles in the development and formation of AS. The vascular endothelium is essential to AS, as it can regulate vascular tension, the proliferation of VSMCs, and vascular permeability (2). ECs also exhibit anti-coagulant and fibrinolysis-promoting capabilities, which can reduce platelet aggregation and immune cell adhesion, preventing the development of pathogenic thrombi (3). Oxidized low-density lipoprotein (ox-LDL) damages ECs in the early stages of AS, causing themsecrete a large amount of chemokines and high expression of endothelial adhesion cytokines (2). Furthermore, ECs can attract monocytes into the endangium, where they differentiate into macrophages, resulting in a partial inflammatory response. Subsequently, VSMCs migrate from the mesomembrane to the subintimal space and proliferate massively (4). This is followed by increased extracellular matrix secretion, which finally leads to the formation of a fibrous plaque cap (5). As lipid build-up increases, macrophages die and create a necrotic core (6). The necrotic core is covered by a fibrous cap, which is composed of oxidized lipoproteins, cholesterol crystals, and cellular debris, with varying levels of remodeling and calcification. (7). In addition, macrophages, when activated by ox-LDL, can transform into cholesterol-rich foam cells, producing matrix metalloproteinases capable of hydrolyzing collagen fibers in the fibrous cap (7). This causes a progressive thinning of the fibrous cap around the necrotic core, which eventually transforms into a vulnerable plaque. Therefore, a favorable control of the roles of ECs, VSMCs, and macrophages is crucial for the prevention and treatment of AS.

**Figure 1:**
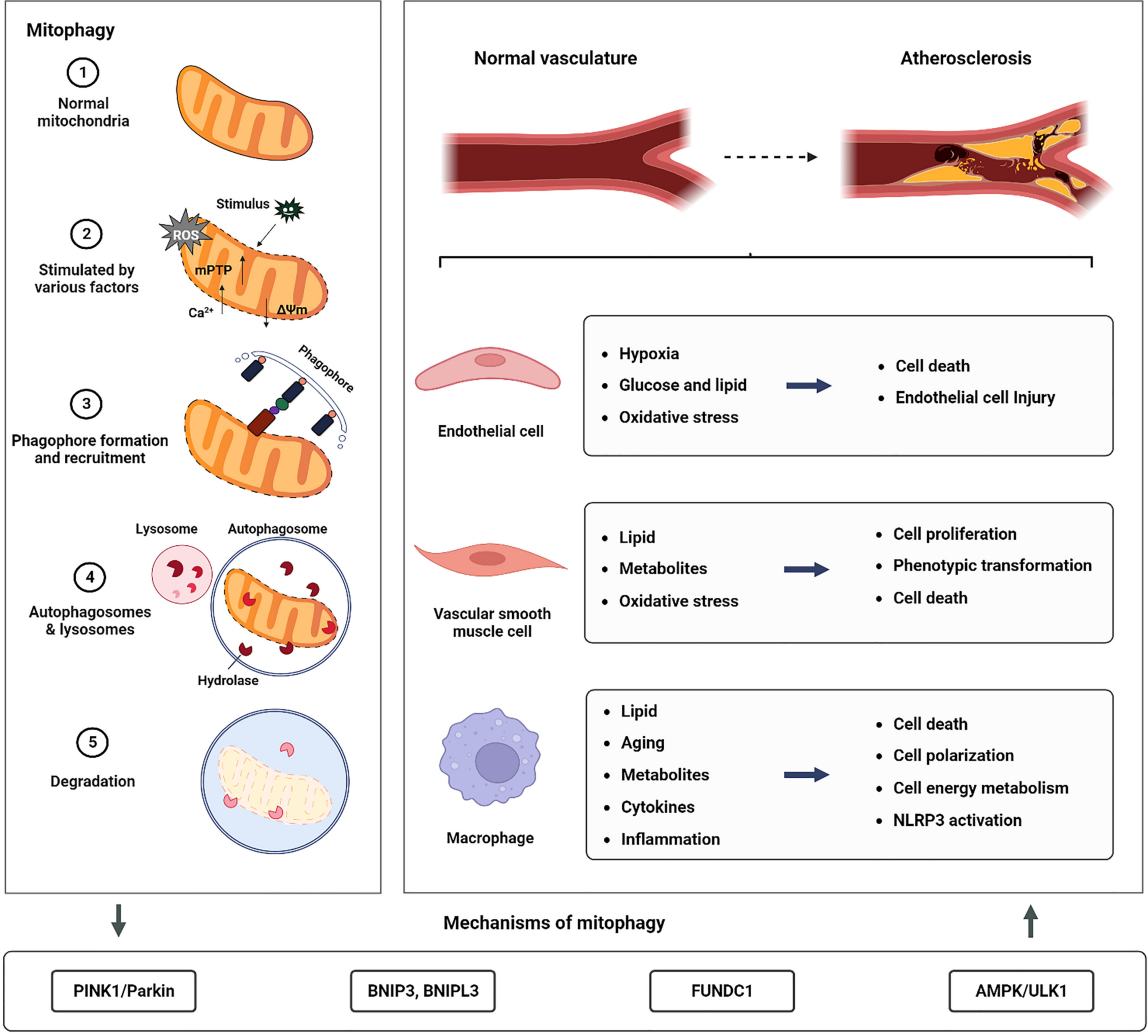
The relationship between mitophagy and AS. The left panel of the image illustrates the macro process of mitophagy. The figure below illustrates the main mitophagy pathway involved in the formation and development of AS. To the right of the figure, the stress conditions and cellular responses reported in current studies on mitophagy in macrophages, ECs, and VSMCs, which are involved in the formation and development of AS are presented.

Mitochondria are organelles found in eukaryotic cells that use oxidative phosphorylation to produce adenosine triphosphate (ATP) and supply energy to the cells (8). Dysfunctional mitochondria, which are a major source of oxidative stress, cause a build-up of excessive reactive oxygen species (ROS) (9, 10). Lemasters et al. (11) developed the idea of “mitophagy” in 2005 and established that mitophagy may selectively remove damaged mitochondria and that this process plays a key role in maintaining mitochondrial homeostasis and lowering ROS generation. The brief process of mitophagy is shown in **Figure 1**. Under physiological conditions, mitophagy can effectively remove mitochondria with impaired functions, maintain intracellular calcium homeostasis, cell signal transduction, and ATP synthesis, and reduce the level of ROS and oxidative stress damage (12). Under stress conditions (such as nutrient starvation, traumatic injury, or ischemia/hypoxia), deficient mitophagy leads to a build-up of poor-functioning mitochondria, resulting in excessive ROS (13, 14). This further damages mitochondria, causing the release of pro-apoptotic proteins into the cytoplasm, exacerbating partial inflammatory responses, and eventually causing the rupture of unstable AS plaques (15, 16). In actuality, the key cells involved in the formation and development of AS depend on mitophagy to operate normally (17–19). As a result, in this review, we outline the current knowledge on the classical mechanism of mitophagy in AS on the role of these cell types.

## 2 Mechanisms of mitophagy

### 2.1 PINK1/Parkin-mediated mitophagy

#### 2.1.1 Regulation of PINK1 stability in polarized mitochondria

It was discovered as early as 2006 that in *Drosophila*, abnormalities in the phosphatase and tensin homolog-long (PTEN)-induced putative kinase protein PINK1 or the Parkin RBR E3 ubiquitin-protein ligase cause mitochondrial dysfunction, muscle degeneration, and decreased longevity (20, 21). PINK1/Parkin-mediated mitophagy may control cell survival or death by eliminating damaged mitochondria (22–24). Under normal conditions, PINK1 enters the mitochondria through the translocase of the outer mitochondrial membrane (TOMM) complex, which is found in the outer mitochondrial membrane (OMM), and the translocase of the inner mitochondrial membrane 23 (TIMM23), and then moves on to the inner mitochondrial membrane (IMM) translocase complex (25). Subsequently, mitochondrial processing peptidase (MPP) in the mitochondrial matrix cleaves the N-terminal mitochondrial-targeting sequence of PINK1, and presenilin-associated rhomboid-like protein (PARL) in the inner mitochondrial matrix cleaves the M-segment of PINK1 (26, 27). The remaining PINK1, with an unstable amino acid at the N-terminus, is released into the cytosol, where it is destroyed by the ubiquitin-proteasome system (UPS) after being targeted by an N-degron2-type E3 ubiquitin ligase (28–30). Due to its degradation, PINK1 remains at very low levels in polarized mitochondria. In addition, Cristina et al. (31) have found that the degradation of PINK1 by the proteasome depends on valosin-containing protein (VCP) and the components of the endoplasmic reticulum-related degradation pathway, such as E3 ligase GP78 and HRD1. However, this does not rule out the possibility that PINK1 serves some sort of physiological purpose.

When mitochondria are damaged, the membrane potential (ΔΨm) of the IMM is depolarized, rendering these mitochondria unable to import PINK1 into the IMM (32), preventing PINK1 from being cleaved by PARL (33). This uncleaved PINK1 is stabilized and accumulates on the OMM, becoming a dimer and attaching to the TOMM complex to limit the import of freshly produced proteins into the mitochondria (29). The TOMM complex supports the autophosphorylation of dimeric PINK1 and aids in its proper localization, both of which are necessary for the recruitment of Parkin and the completion of mitophagy (34). Furthermore, TOMM7 (35), the complex subunit of mitochondrial contact-site and cristae organizing system (36), and the mitochondrial protease OMA1 (35) are involved in controlling PINK1’s stability on the outer membrane.

#### 2.1.2 Recruitment of Parkin and phosphorylation of ubiquitin

PINK1 at the OMM is activated by autophosphorylation at Ser228 (37) and Ser402 (38), and it affects Parkin in two ways. First, PINK1 can phosphorylate ubiquitin (UB) attached to OMM proteins at Ser65 in the mitochondria (39). Due to the high affinity of Parkin for phosphorylated ubiquitin (pSer65-UB), this can drive its translocation from the cytosol to the mitochondria (40, 41). Furthermore, PINK1 directly phosphorylates Parkin at Ser65 in the UB-like domain and attracts Parkin from the cytoplasm to the OMM to recruit autophagy mechanisms (30, 39, 42). In both cases, the activated Parkin conjugates more UB to OMM-associated proteins, which are then phosphorylated by PINK1. This forms a positive feedback loop that amplifies the initial signal, leading to extensive recruitment and ubiquitination of Parkin and the decoration of the OMM with pSer65-UB (43, 44). In addition, the study of crystal structures has provided important insights into the interaction between PINK1 (45, 46), Parkin (47–49), and UB (50). Studies have shown that phosphorylated UB (pUB) can bind two distinct sites on the Parkin structure. The high-affinity site on RING1 controls the Parkin localization, and the low-affinity site on RING0 releases the Parkin autoinhibition (51). The interaction between Parkin’s RING1 and pUB results in the disengagement of the UB-like domain from the core structure and the liberation of Parkin from inhibitory interactions and accumulation on OMM (52, 53). Notably, the UB-like domain of Parkin, when replaced with UB, is more readily activated by PINK1 phosphorylation (51). It can be seen that pUB holds an important role in the feedforward mechanism because it is not only the signal of Parkin recruitment but also the signal of activation.

Parkin is activated by the recruitment and phosphorylation of PINK1, which leads to the ubiquitination of various substrates at the OMM, such as voltage-dependent anion-selective channel protein 1 (VDAC1), mitofusin 1 (MFN1), and mitofusin 2 (MFN2). This results in the general ubiquitination of the mitochondria (54). Indeed, VDAC1 plays a crucial role in regulating mitophagy and apoptosis as a key substrate of Parkin. VDAC1 deficient in polyubiquitination impedes mitophagy, while VDAC1 deficient in monoubiquitination promotes apoptosis by increasing mitochondrial Ca^2+^ uptake through the mitochondrial calcium uniporter channel (55, 56). Pharmacological inhibition of VDAC1 significantly prevents apoptosis induced by sonodynamic therapy combined with endogenous protoporphyrin IX derived from 5-aminolevulinic acid in human myeloid leukemia-derived (THP-1) monocytes by decreasing Ca^2+^ levels and oxidative stress (57).

#### 2.1.3 Mitophagy adaptors

**Figure 2:**
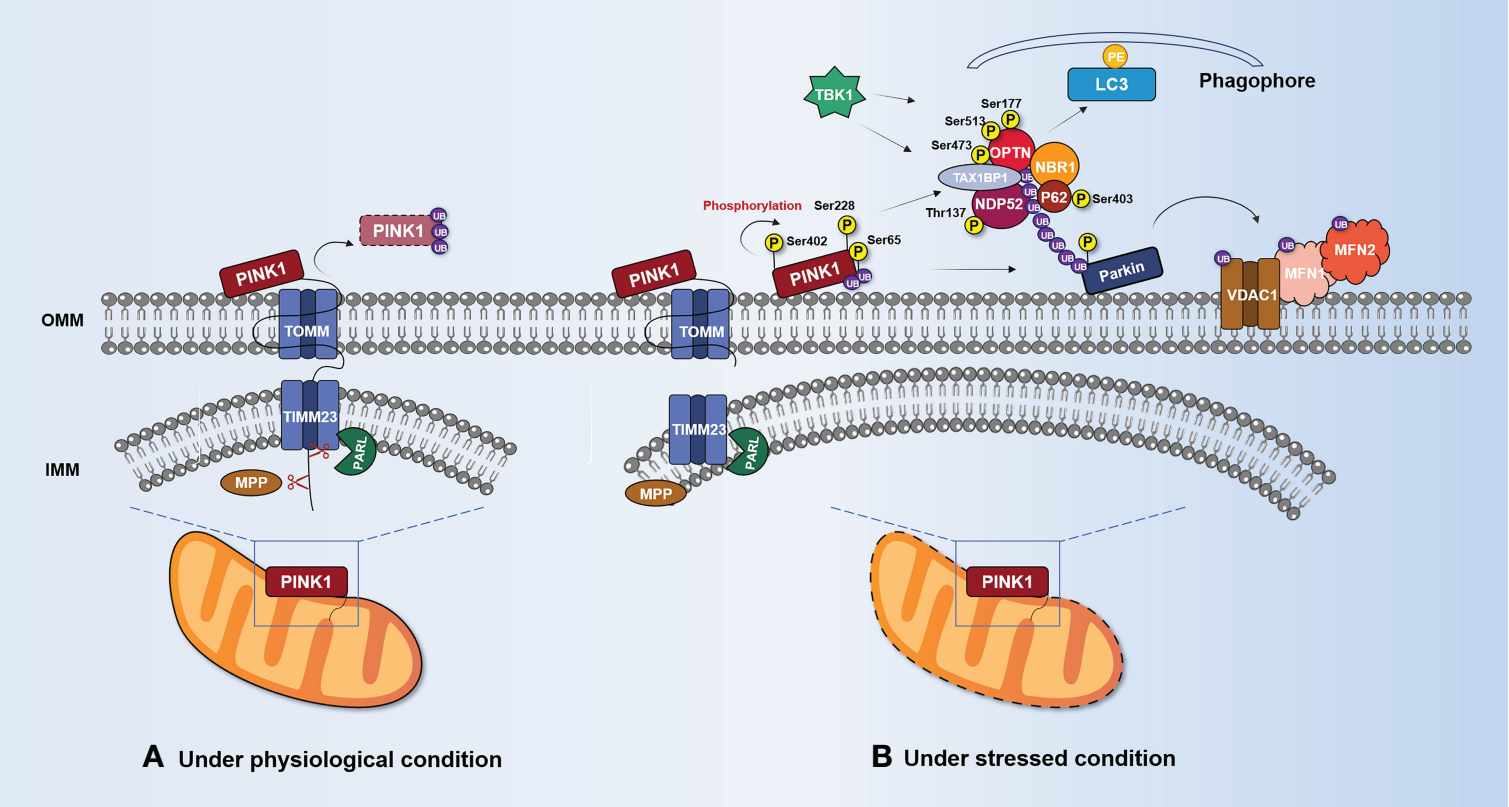
PINK1/Parkin-mediated mitophagy. **(A)** Under normal circumstances, PINK1 enters the mitochondria *via* TOMM and TIMM23. MPP and PARL cleave PINK1. The UPS degrades the remaining portion after it is released into the cytosol. **(B)** Under stress conditions, PINK1 accumulates and stabilizes on the OMM, forming dimers and interacting with TOMM complexes. Accumulated PINK1 (Ser228 and Ser402) is phosphorylated on its own and activated, phosphorylating Ser65 to recruit UB and Parkin. OMM protein substrates (VDAC1, MFN1, and MFN2) are ubiquitinated by activated Parkin. Mitophagy adaptors (p62, OPTN, NDP52, and NBR1) are recruited by the polyubiquitin chain linked to Parkin to start the process of delivering mitochondria to autophagosomes. These mitophagy adaptors work together with LC3 to enclose damaged mitochondria in autophagosomes. TBK1 can promote mitophagy by phosphorylating mitophagy adaptors such as p62 (Ser403), OPTN (Ser177, Ser473, and Ser513), and NDP52 (Thr137). IMM, inner mitochondrial membrane; LC3, microtubule-associated protein 1 light chain 3; MFN1, mitofusin 1; MFN2, mitofusin 2; MPP, mitochondrial processing peptidase; NBR1, neighbor of BRCA1 gene 1; NDP52, nuclear domain 10 protein 52; OMM, outer mitochondrial membrane; OPTN, optineurin; Parkin, E3 ubiquitin-protein ligase Parkin; PARL, presenilin associated rhomboid-like protein; PE, phosphatidyl ethanolamine; PINK1, serine/threonine-protein kinase PINK1; SQSTM1/p62, sequestosome-1/ubiquitin-binding protein p62; TBK1, tank-binding kinase 1; TIMM23, inner mitochondrial membrane 23 translocase; TOMM, outer mitochondrial membrane translocase; UB, ubiquitin; VDAC1, voltage-dependent anion-selective channel protein 1.

The Atg8/LC3 family of autophagy-related proteins is a component of the autophagosome membrane that functions as a bridge between cargo and autophagosomes (58). During autophagy activation, Atg8 family proteins bind to phosphatidyl ethanolamine (PE) through a lipidation process (59). The newly synthesized Atg8 is cleaved by Atg4 family members and then conjugated to PE through a ubiquitination-like reaction cascade (60–62). Once inserted into autophagic membranes, the lipidized LC3 serves as a docking site for various autophagy adaptors and receptors that facilitate the movement of autophagic vesicles and target selection, respectively (63). After the remodeling of the OMM through proteasomal degradation of partially ubiquitinated proteins such as VADC1, MFN2, and MFN1, UB-bound adaptor proteins are recruited to form a substrate poly-UB chain (64, 65). This chain causes the UB-binding protein p62, optineurin (OPTN), nuclear domain 10 protein 52 (NDP52), Tax1-binding protein 1 (TAX1BP1), and the BRCA1 gene 1 protein (NBR1) to aggregate and bind to ubiquitinated proteins (66). Notably, OPTN and NDP52 are the primary, yet redundant, receptors for Parkin-mediated mitophagy, whereas p62 and NBR1 are dispensable (66). Unlike other autophagy receptors for mitochondria, p62 preferentially localizes to the domain between adjacent mitochondria, and aggregates damaged mitochondria using its polymerase basic protein oligomerization domain but does not regulate LC3 recruitment to mitochondria (67, 68). The UB-binding domain of autophagy adaptors recognizes mono- or polyubiquitin chains conjugated to proteins at the surface of the mitochondrion, while the LC3 interaction region (LIR) interacts with Atg8 family proteins. Subsequently, autophagosomes engulfing damaged mitochondria fuse with lysosomes to form “autolysosomes” that degrade the cargo (69, 70). It is worth noting that PINK1, as both a sensor and an effector of mitochondrial damage, can recruit NDP52 and OPTN to damaged mitochondria without the assistance of Parkin (66). Furthermore, tank-binding kinase 1 (TBK1) can phosphorylate some autophagy adaptors, which increases the binding affinity of those proteins to UB chains and Atg8/LC3 proteins (71, 72). TBK1 phosphorylates OPTN (Ser177) (73), NDP52 (Thr137) (74), and p62 (Ser403) (72) to enhance their LC3-binding affinity. In addition, OPTN (Ser473 and Ser513) (73, 75) and p62 (Ser403) (72) are phosphorylated by TBK1, increasing their ability to bind to UB chains, as shown in **Figure 2**. It is worth noting that PINK1’s phosphorylation of UB is necessary to recruit various autophagy adaptors to mitochondria (66, 76). Mitochondrial damage triggers the phosphorylation of TBK1 (Ser172), which activates its catalytic activity in a PINK1/Parkin-dependent manner (77). This suggests that the assembly of UB chains by Parkin at the OMM may promote a feedforward loop that drives TBK1 phosphorylation to enhance adaptor binding to UB chains.

#### 2.1.4 Deubiquitinases and PTEN-L as regulators of mitophagy

Recent studies have found that phosphatase and tensin homolog-long (PTEN-L) can effectively prevent the mitochondrial translocation of Parkin, dephosphorylate pSer65-UB, and reduce the level of Parkin phosphorylation (78). This activity keeps Parkin in an autoinhibited state, inhibits its E3 ligase activity, and prevents its recruitment and activation at the OMM. This leads to a blockade of the feedforward mechanism and inhibition of mitophagy (78, 79).

Deubiquitinases (DUBs) are members of the cysteine and metalloproteinase families, which cleave UB from protein substrates. Multiple DUBs regulate mitophagy positively or negatively. USP8 has been demonstrated to selectively remove K6-linked UB chains from Parkin and promote Parkin localization to depolarized mitochondria (80, 81). Conversely, USP15, USP30, and USP35 have been shown to remove Parkin-mediated ubiquitination of OMM proteins and attenuate subsequent depolarized mitochondrial clearance (82–84). Among these DUBs, USP30 tends to hydrolyze unanchored and mitochondrially conjugated Lys6- and Lys11-linked UB chains, delaying or antagonizing Parkin-mediated mitophagy by interfering with its recruitment to mitochondria (85–88). Notably, USP14 is one of the major deubiquitinating enzymes that associates with the proteasome, and its primary function is believed to be preventing proteins from being misubiquitinated *in vivo* by cleaving UB chains on protein substrates, thereby participating in the process of regulated protein degradation (89, 90). Studies have shown (91) that USP14 is highly expressed in human aortic valve tissue, which may be related to its inhibition of the degradative activity of the proteasome. AS is a lipid-inflammatory process. USP14 can activate the non-canonical NF-κB signaling pathway and promote the expression of tumor necrosis factor (TNF)-α, interleukin (IL)-6, and IL-18, which interact to form a positive feedback cycle, promote lipid deposition, and ultimately induce the occurrence of AS (92, 93). Fu et al. (94) have shown that the overexpression of USP14 in ECs can interfere with the activation of NF-κB, and adenovirus injection of USP14 can alleviate AS in ApoE^–/–^ mice. Inhibition of USP14 reduces the formation of foam cells in AS by down-regulating CD36-mediated lipid uptake (95). Furthermore, the knockdown of USP14 prevents the proliferation of VSMCs induced by platelet-derived growth factor (PDGF)-BB, by inhibiting the mTOR complex 1 (mTORC1)/ribosomal protein S6 kinase signal pathway (96). Nonetheless, it remains unclear how OMM-associated poly-UB prevents cleavage by DUBs. One study found that the PINK1-dependent phosphorylation of UB rendered ubiquitinated OMM proteins DUB-resistant (97). Collectively, understanding the function of DUBs and their regulation in cellular mitophagy may provide a novel and effective approach to the treatment of AS and is worthy of further study.

### 2.2 BNIP3L and BNIP3-mediated mitophagy

#### 2.2.1 BNIP3L/Nix

BCL2-interacting protein 3-like (BNIP3L/Nix) is an OMM protein that belongs to the BCL2 family of BH3-only proteins. Studies have demonstrated (98) that BNIP3L is a mitophagy receptor that may bind LC3 *via* the LIR motif at its N-terminus. Notably, BNIP3L can inhibit ox-LDL-induced ROS production and nucleotide oligomerization domain (NOD)-like receptor thermoprotein dome-associated protein 3 (NLRP3) inflammasome activation in macrophages through mitophagy (99). In addition, dynamic phosphorylation can regulate BNIP3L-mediated mitophagy. Phosphorylation of Ser17, Ser34, Ser35, and other residues near the LIR domain improves the interaction between the BNIP3L and LC3 homologs, targeting mitochondria into autophagosomes for degradation (100, 101).

Protein kinase PRKA phosphorylates BNIP3L at Ser212, causing it to be exported from mitochondria and sarcoplasmic sites to the cytosol, preventing mitophagy (102). Furthermore, the dimerization of BNIP3L confers greater mitophagy receptor activity than the monomeric form (103). Notably, glycines 204 and 208 in the transmembrane domain of BNIP3L are important for BNIP3L dimerization, whereas phosphorylation of C-terminal BNIP3L disrupts dimerization (103). Unfortunately, it remains unclear how BNIP3L dimers trigger mitophagy and the molecular mechanism underlying BNIP3L dimerization.

#### 2.2.2 BNIP3

BCL2-interacting protein 3 (BNIP3) mRNA is positively correlated with necrotic core size in human AS plaques (104). In its role as a mitophagy receptor, BNIP3 is typically expressed as an inactive monomer in the cytosol. Upon hypoxia, it forms stable homodimers through its C-terminal transmembrane domain and is integrated into the OMM through this domain (103, 105). However, mutations can disrupt homodimerization and cause mitophagy defects (106). Upon autophagy activation, BNIP3 causes mitophagy by triggering mitochondrial depolarization and subsequent sequestration of mitochondria into autophagic bodies (107). Reduced BNIP3 expression in mouse embryonic stem cells can cause the accumulation of abnormal mitochondria, along with decreased ΔΨm, increased ROS production, and decreased ATP production (108). The BNIP3 gene promoter contains a hypoxia-inducible factor (HIF) response element, and HIF-1 promotes the expression of BNIP3 during hypoxia, which limits ROS production *via* mitophagy (109–111).

**Figure 3:**
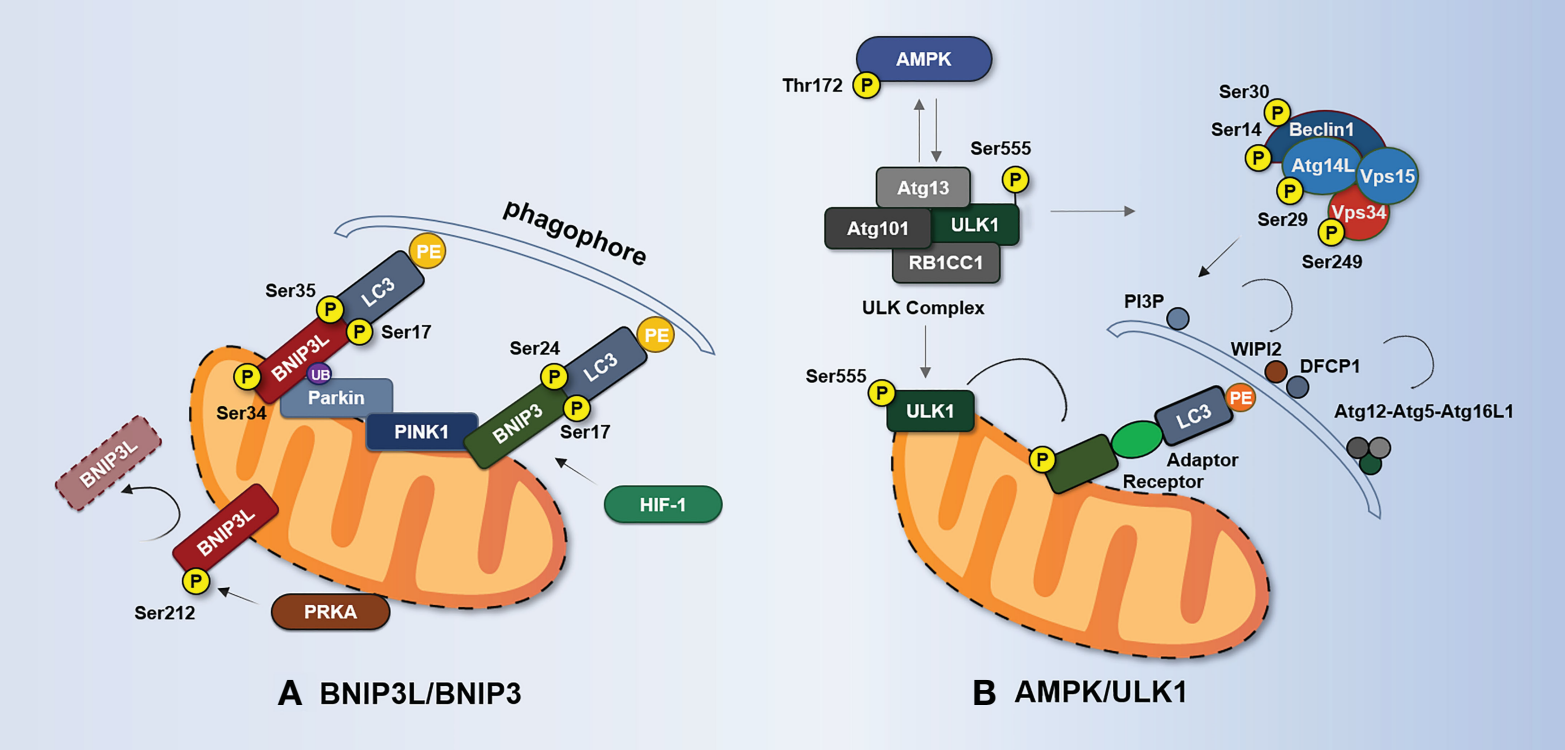
BNIP3L-/BNIP3-mediated and AMPK/ULK1-mediated mechanisms of mitophagy. **(A)** Phosphorylation of BNIP3L (Ser17, Ser34, and Ser35) enhances the interaction between BNIP3L and LC3 homologs. PRKA phosphorylates BNIP3L at Ser212 and causes BNIP3L to leave the mitochondria, thereby hindering mitophagy. Phosphorylation at Ser17 and Ser24 flanking the LIR domain of BNIP3 enhances the BNIP3–LC3 interaction and promotes mitophagy. Hif-1α enhances mitophagy by promoting the expression of BNIP3 during hypoxia. BNIP3L can be regulated by Parkin ubiquitination. BNIP3 interacts with PINK1 and promotes PINK1 accumulation on OMM. **(B)** When activated by AMPK phosphorylation, ULK1 recruits ULK complexes consisting of ULK1, Atg13, RB1CC1, and Atg101. The activated ULK complex recruits the Beclin1–Atg14L–Vps34–Vps15 complex. This complex is responsible for the production of Ptdlns3P and the formation of omegasomes at autophagosome formation sites and the recruitment of WIPI2 and DFCP1. WIPI2 and DFCP1 recruit and bind the complex of Atg16L1–Atg5–Atg12 and mediate covalent modification of PE and Atg8/LC3. ULK1 directly phosphorylates Beclin1 at Ser14 and Ser30, Atg14L at Ser29, and Vps34 at Ser249. Under stress, AMPK phosphorylates Ser555 of ULK1, and phosphorylated ULK1 in turn reduces the phosphorylation of Thr172 of the AMPK α-subunit. Phosphorylated ULK1 also phosphorylates the receptor regulating mitophagy. AMPK, adenosine 5′-monophosphate-activated protein kinase; Atg, autophagy-related protein; BNIP3, BCL2-interacting protein 3; BNIP3L/Nix, BCL2-interacting protein 3-like; DFCP1, double FYVE-containing protein 1; RB1CC1, RB1-inducible coiled-coil protein 1; HIF-1, hypoxia-inducible factor 1; PI3P, phosphatidylinositol 3-phosphate; PRKA, protein kinase A; ULK1, unc-51-like autophagy-activating kinase 1; Vps, vacuolar protein sorting-associated protein; WIPI2, WD repeat domain phosphoinositide-interacting protein 2.

Similar to BNIP3L, the interaction between BNIP3 and LC3 is also regulated by phosphorylation (112, 113), as shown in **Figure 3A**. Zhu et al. (101) have found that phosphorylation at Ser17 and Ser24 on both sides of the LIR domain of BNIP3 enhances BNIP3–LC3 interaction and promotes mitophagy. Further studies have revealed that unc-51-like autophagy-activating kinase 1 (ULK1) is responsible for the phosphorylation of Ser17 in BNIP3 and of Ser35 in BNIP3L, whereas TBK1 may be required for the phosphorylation of Ser24 and Ser34 in BNIP3 and BNIP3L, respectively (114).

#### 2.2.3 Relationship between BNIP3/BNIP3L and PINK1/Parkin-dependent mitophagy

BNIP3 and BNIP3L may be involved in PINK1/Parkin-mediated mitophagy. According to Onishi et al. (115, 116), Parkin may ubiquitinate BNIP3L to attract NBR1, a specific autophagic adaptor of LC3, to mitochondria, causing autophagosomes to form around damaged mitochondria. This study demonstrated that BNIP3L acts as a substrate for Parkin and that mitochondrial clearance induced by BNIP3L depends on the presence of Parkin. In addition, Zhang et al. (117) have found that BNIP3 interacts with PINK1 and promotes PINK1 accumulation at the OMM, leading to Parkin translocation to mitochondria. In conclusion, BNIP3 and BNIP3L can trigger mitophagy through various signaling pathways and cytokines (103). Loss of mitophagy speeds up cell death, but its relationship with AS is poorly understood, necessitating further research on the kinases and phosphatases of BNIP3 and BNIP3L in this context.

### 2.3 FUNDC1-mediated mitophagy

#### 2.3.1 Phosphorylation and dephosphorylation of FUNDC1

**Figure 4:**
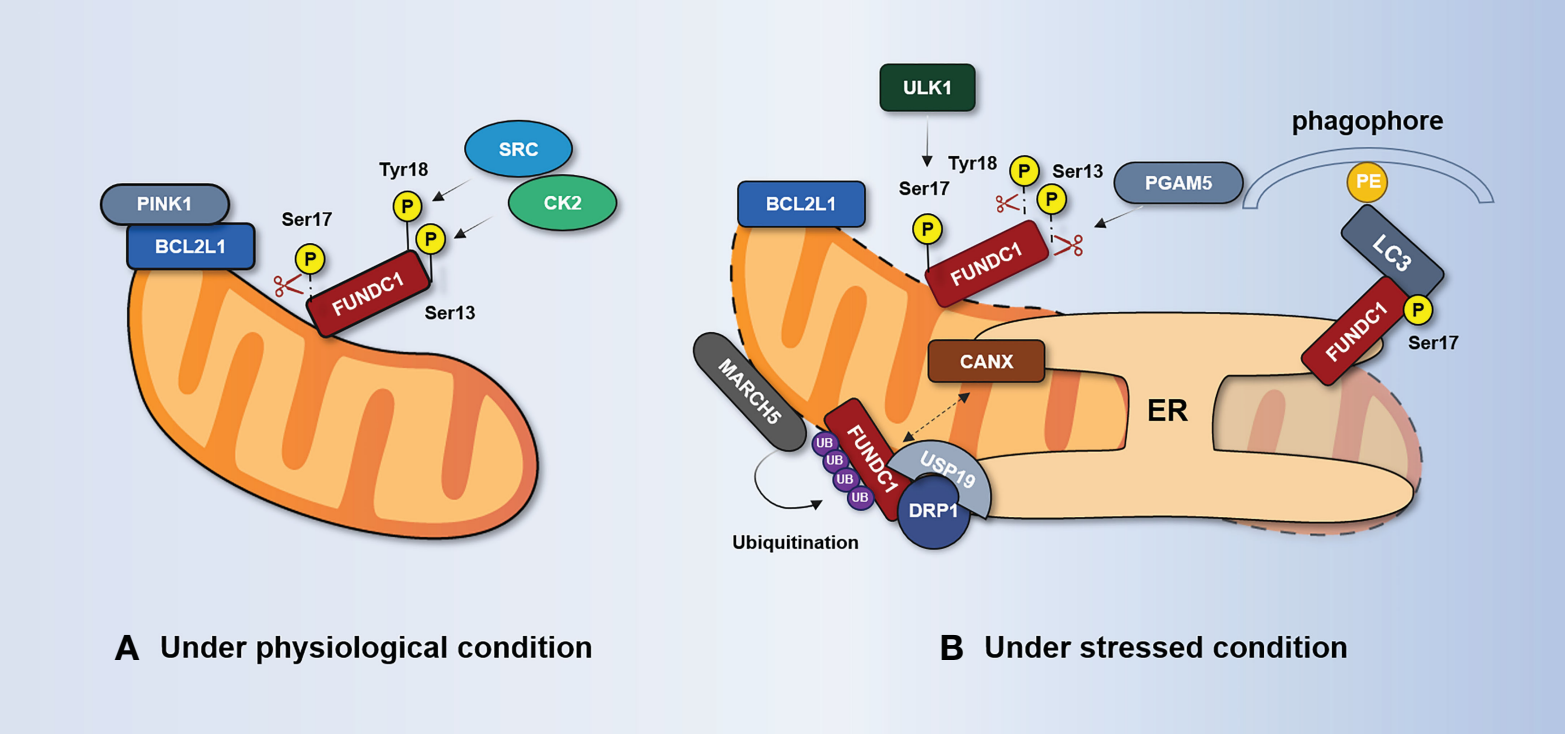
FUNDC1-mediated regulation of mitophagy. **(A)** Under physiological conditions, FUNDC1 is phosphorylated at Ser13 by CK2, phosphorylated at Tyr18 by SRC, and dephosphorylated at Ser17. It suppresses the interaction of FUNDC1 with LC3. BCL2L1 inhibits PGAM5 activity through the BH3 domain and prevents dephosphorylation at Ser13. **(B)** Under stress conditions, PGAM5 dissociates from BCL2L1 and dephosphorylates Ser13, thereby promoting FUNDC1-mediated mitophagy. The kinase ULK1 translocates to the mitochondria and phosphorylates FUNDC1 at Ser17. The separation of CANX from FUNDC1 and the incorporation of deubiquitinated FUNDC1 by the deubiquitinating enzyme USP19 at the MAM junction enhances the interaction between DRP1 and FUNDC1. BCL2L1, Bcl-2 like protein 1; CANX, calnexin; CK2, casein kinase 2; DRP1, dynamic-related protein 1; ER, endoplasmic reticulum; FUNDC1, FUN14 domain-containing protein 1; MARCH5, mitochondrial E3 ubiquitin ligase MARCH5; PGAM5, PGAM family member 5; SRC, SRC proto-oncogene; non-receptor tyrosine kinase; USP, ubiquitin-specific protease.

FUN14 domain-containing protein 1 (FUNDC1) is a mitophagy receptor in mammals. Phosphorylation and dephosphorylation of Ser17, Ser13, and Tyr18 in FUNDC1’s LIR domain can have a direct impact on the FUNDC1–LC3 interaction mechanism (118, 119). Casein kinase 2 (CK2) protein kinase, SRC proto-oncogene, non-receptor tyrosine kinase (SRC), and ULK1 are the main regulators of FUNDC1 phosphorylation, while mitochondrial phosphatase PGAM family member 5 (PGAM5) and Bcl-2 like protein 1 (BCL2L1) are considered key regulators of FUNDC1 dephosphorylation (120–122), as shown in **Figure 4**. FUNDC1 can exist in OMM under normal physiological conditions without mediating mitophagy (123). Furthermore, FUNDC1 is phosphorylated and has little mitophagy activity. FUNDC1 is phosphorylated by CK2 kinase at Ser13 and SRC kinase at Tyr18, which reduces the binding of FUNDC1 and LC3, thereby preventing mitophagy (119, 124). However, under hypoxia, the dephosphorylation of FUNDC1 at Tyr18 and the dephosphorylation of Ser13 by PGAM5 phosphatase both enhance the interaction between FUNDC1 and LC3, which further leads to the selective removal of dysfunctional mitochondria (123). On the other hand, dephosphorylation of FUNDC1 at Ser17 inactivates FUNDC1, but it is phosphorylated by ULK1 kinase under hypoxia or mitochondrial uncoupled stimulation, which increases the binding to LC3 and promotes mitophagy (125).

It is worth noting that the regulation of FUNDC1-mediated mitophagy is also influenced by the dephosphorylation and phosphorylation of other proteins. In normoxic conditions, BCL2L1 can interact with PGAM5 through the BH3 domain and inhibit PGAM5 activity, preventing the dephosphorylation of Ser13 (126). During hypoxia, BCL2L1 is degraded, releasing PGAM5 to promote Ser13 dephosphorylation, thereby initiating or enhancing FUNDC1-mediated mitophagy (120, 127). Meanwhile, the kinase ULK1 is recruited to mitochondria and phosphorylates FUNDC1 at Ser17, enhancing its interaction with LC3 to promote mitophagy (125). However, the regulatory mechanism of dephosphorylation of FUNDC1 at Tyr18 remains unclear. These findings highlight the complex and intricate nature of FUNDC1-mediated mitophagy, and further research is needed to fully understand its regulation and signaling pathways.

#### 2.3.2 Phosphorylation-independent mechanisms of FUNDC1 regulation

Recent research has demonstrated that FUNDC1’s negative regulation of phosphorylation-independent activity plays a significant role in mitophagy. Mitochondria-associated endoplasmic reticulum (MAM) is a dynamic membrane-coupling region formed by the coupling of the OMM and the ER and is involved in mitochondrial dynamics, mitophagy, Ca^2+^ exchange, and other processes (128). FUNDC1 mediates the formation of the MAM and accumulates at the MAM, promoting mitochondrial activity through indirect binding to calnexin (CANX) on the ER (129). A high expression of FUNDC1 promotes MAM formation, leads to increased cytosol Ca^2+^ levels, promotes phosphorylation of serum response factor, and enhances its binding to the vascular endothelial growth factor receptor 2 (VEGFR2) promoter (130). As a result, angiogenesis is encouraged and VEGFR2 expression is elevated.

Wu et al. (129) found that the association between FUNDC1 and CANX disappears in the late stage of hypoxia, and FUNDC1 mainly interacts with dynamic-related protein 1 (DRP1), which causes DRP1 to initiate mitochondrial fission and mitophagy at MAMs. Interestingly, the deubiquitinating enzyme USP19 incorporates deubiquitinated FUNDC1 at the MAM junction, which enhances the interaction between DRP1 and FUNDC1 to promote mitophagy and mitochondrial fission (131). This suggests that FUNDC1 might serve as a bridging link between mitophagy and mitochondrial dynamics. Furthermore, Chen et al. (132) have discovered that ubiquitination of FUNDC1 at K119, mediated by the mitochondrial E3 ubiquitin ligase MARCH5, could inhibit hypoxia-induced mitophagy *via* the proteasomal degradation of FUNDC1 during hypoxia.

#### 2.3.3 FUNDC1 and angiogenesis

In the context of AS, neoangiogenesis may increase the flow of local nutrients and oxygen, promoting the progress and remodeling of plaques (133, 134). However, due to the vulnerability of the neovasculature, this may lead to plaque instability, rupture, and thromboembolism (135, 136). Wang et al. (130) have found that FUNDC1-specific deletion in ECs disrupts ECs’ ability to form MAMs and lowers the expression of VEGFR2, which inhibits the growth of functional blood vessels both *in vitro* and *in vivo*. However, Wang et al. (137) have discovered that FUNDC1 silencing impairs mitophagy flux and worsens angiotensin II (Ang II)-induced VSMC proliferation and migration. Overexpression of FUNDC1 inhibits Ang II-induced proliferation and migration of VSMCs, blocking Ang II-induced expression of cell adhesion cytokines and matrix metalloproteinase-9, increasing ΔΨm, and reducing intracellular ROS production (137). These findings suggest that FUNDC1 has different effects on participation in angiogenesis in different cell types and more research is needed to fully understand its role in AS.

### 2.4 AMPK/ULK1-mediated mitophagy

#### 2.4.1 Role of ULK1 in autophagosome formation

As previously mentioned, ULK complexes and Atg9 are required for the formation of mitochondrial autophagosomes (138). ULK1, Atg13, RB1-inducible coiled-coil 1 (RB1CC1), and Atg101, which can be activated by adenosine 5′-monophosphate-activated protein kinase (AMPK) phosphorylation, are the components of the ULK complex (139). The activated ULK complex translocates to the ER tubule vesicle region labeled by Atg9 vesicles and recruits the Beclin1–Atg14L–vacuolar protein sorting-associated protein (Vps) 34–Vps15 complex (140). This complex is responsible for the production of phosphatidylinositol 3-phosphate (PI3P) at the autophagosome formation site and the formation of omegasomes (141–143). Omegasomes amplify local PI3P signaling to attract members of the WIPI protein family, such as WD repeat domain, phosphoinositide interacting 2 (WIPI2), and double FYVE-containing protein 1 (DFCP1) (144, 145). These proteins then attract and bind the Atg16L1–Atg5–Atg12 complex, which is responsible for the covalent modification of PE and Atg8/LC3 (146). This is a prerequisite for the formation of autophagosomes. Notably, ULK1 directly phosphorylates Beclin1 at Ser14 and Ser30 (147, 148), Atg14L at Ser29 (149), and Vps34 itself at Ser249 (148), thereby enhancing Vps34 activity, PI3P production, and initiation of autophagy.

#### 2.4.2 The AMPL/ULK1 axis signaling cascade

AMPK is a metabolic and redox state sensor that is widely expressed in various tissues and organs. It consists of one catalytic subunit (α-subunit) and two regulatory subunits (a β-subunit and a γ-subunit). AMPK senses the energy available in cells by binding directly to adenine nucleotides, and is activated in response to changes in the ratio of ATP to ADP or ATP to AMP in cellular energy (150–152). AMPK plays a crucial role in regulating cellular metabolism by shifting metabolism toward increased catabolism and decreased anabolism through phosphorylation of key proteins in various pathways, such as mTORC1 (153), lipid homeostasis (154, 155), and glycolysis (156, 157). In addition, AMPK is involved in various physiological processes, such as metabolism, cytoskeleton assembly, transcriptional control, apoptosis, and autophagy (158, 159). Zhang et al. (160) have shown that AMPKα1 deficiency impairs autophagy-mediated macromonocyte differentiation and decreases macrophage survival, attenuating AS in ApoE^–/–^ mice. Furthermore, Chen et al. (161) believe that chronic exercise training may improve aortic endothelial and mitochondrial function by increasing the expression of AMPKα2, decreasing the expression of BNIP3L and LC3B, and increasing aortic mitochondrial content.

Recent studies have shown that the AMPK/ULK1 axis is critical for selective mitochondrial clearance (162, 163). Cells deficient in ULK1 or AMPK show an increased accumulation of damaged mitochondria (164–166). Hypoxia significantly induces AMPKα1 phosphorylation at Thr172 and phosphorylation of ULK1 at Ser555 and Ser317, whereas phosphorylation of ULK1 at Ser757 is decreased (163). This may be due to the enhanced combination of AMPKα1 and ULK1, while inhibiting or silencing AMPKα leads to the loss of ULK1 phosphorylation at Ser555 and interferes with the translocation of ULK1 to mitochondria (163). Furthermore, under glucose starvation, AMPK promotes autophagy by directly phosphorylating ULK1 at Ser317 and Ser777 (167). Under nutrient-sufficient conditions, high activity of mTOR prevents ULK1 activation by phosphorylating Ser757 of ULK1 and inhibiting the interaction between ULK1 and AMPK (167).

Notably, ULK1 regulates AMPK activity by directly phosphorylating all three AMPK subunits (168). This suggests that phosphorylation of ULK1 in turn acts in a feedback loop, as shown in **Figure 3B**. Although there is currently insufficient evidence showing that AMPK/ULK1-mediated mitophagy is relevant to AS, it has been found that translocation of the apoptosis-triggering factor AIF from mitochondria to the nucleus, which induces macrophage cell death, is observed in ULK1-deficient THP-1 cells upon lipopolysaccharide (LPS) plus nigericin stimulation (169). Additionally, a high-protein diet has been shown to induce macrophage apoptosis through increased mTORC1 targets, phosphorylation of ULK1, and concomitant inhibition of autophagosome formation, ultimately promoting the progression of AS plaque (170).

#### 2.4.3 Series of AMPK/ULK1 axis and PINK1/Parkin axis

As mentioned above, the AMPK/ULK1 axis is linked to the FUNDC1, BNIP3, and BNIP3L-mediated mitophagy axes. In fact, the AMPK/ULK1 axis also interacts with the PINK1/Parkin axis. Under hypoxia or glucose or nutrient starvation, AMPK is activated in the cytoplasm and subsequently phosphorylates Ser555 and Ser757 of ULK1 (163). ULK1 activation results in the phosphorylation of Parkin at Ser108 (171). Inhibition of AMPKα1 activation or silencing of AMPKα1 causes loss of ULK1 phosphorylation and interferes with ULK1 translocation to mitochondria. AMPKα1 deficiency impairs autophagy-mediated macromonocyte differentiation and decreases macrophage survival, thereby attenuating AS in ApoE^–/–^ mice (160). Notably, PINK1-mediated phosphorylation of Parkin (Ser65) requires ULK1-dependent phosphorylation of Parkin at Ser108 (171). This highlights the critical and early role played by the AMPK/ULK1 axis in mitophagy. The interaction between the AMPK/ULK1 axis and the PINK1/Parkin axis deserves further investigation.

In addition, the effects of AMPK and ULK1 on the PINK1/Parkin axis may be independent. Overexpression of AMPKα2 can interact with Ser495 - the phosphorylation site of PINK1 - to enhance mitophagy mediated by it (172). Lee et al. (173) have reported that Parkin is an AMPK substrate and can be activated by AMPK phosphorylation at Ser9. As a result, cell death during necrotizing apoptosis is decreased, and receptor-interacting protein kinase 3 (RIPK3) is polyubiquitinated. Likewise, according to Hung et al. (171), ULK1 phosphorylates Parkin at Ser108 and facilitates Parkin translocation to the mitochondria. Inhibition of ULK1 or mutation of this phosphorylation site results in delayed activation of Parkin and defective mitophagy. Thus, as an early mechanism of mitophagy, the AMPK/ULK1 axis is located upstream of many classical mitophagy pathways. In addition, independent of AMPK, the mitophagy adaptors NDP52 and OPTN can also recruit ULK1 (174). In fact, it is well known that the AMPK/ULK1 axis plays an important role in the prevention and treatment of AS. It can prevent oxidative stress and inflammation, control energy metabolism, prevent apoptosis, play anti-proliferation and anti-migration roles in VSMCs, and inhibit vascular calcification, to protect ECs (160, 175, 176). However, further research is necessary to determine whether AMPK/ULK1 can control mitophagy to prevent and treat AS.

### 2.5 Other receptors for mitophagy

In addition to the above pathways, it has been found that some receptors are located in the OMM, such as BECN1-regulated activation molecule in autophagy protein 1 (AMBRA1) (177), Bcl-2-like protein 13 (BCL2L13) (178), and FKBP prolyl isomerase FKBP8 (179). These receptors share a common LIR motif in their N-terminal domain that can interact with LC3B. According to studies, AMBRA1 has the ability to start mitophagy in both Parkin-dependent and Parkin-independent ways. AMBRA1 phosphorylation at Ser1014 in LIR regulates AMBRA1–LC3 interaction and mitophagy activity (180). This motif has the capacity both to enhance Parkin-mediated mitochondrial clearance and to control Parkin-independent mitophagy (181). As a result of damaged mitochondria, choline dehydrogenase also builds up at the OMM and interacts with p62 to trigger the recruitment of LC3 and autophagosomes (182). A recently identified receptor that controls mitophagy is the IMM-localized protein prohibitin 2 (PHB2), which differs from the OMM-localized mitophagy receptor (183, 184). PHB2 depletion can prevent the stabilization of PINK1 and recruitment of Parkin, UB, and OPTN to mitochondria *via* the PARL–PGAM5 axis, resulting in the inhibition of mitophagy upon mitochondrial membrane depolarization or aggregation of misfolded proteins (185). Overexpression of PHB2 directly induces Parkin recruitment to mitochondria. In fact, Parkin can directly bind to PHB2 through its RING1 domain and promote the ubiquitination of PHB2, thereby enhancing the interaction between PHB2 and LC3 (186). In addition, PHB2 may play an important role in maintaining the contractile phenotype of VSMCs by interacting with cartilage oligomeric matrix protein (COMP) and regulating mitochondrial oxidative phosphorylation (187). However, it is not yet known whether the mitophagy receptors on the OMM and IMM act synergistically to promote mitophagy.

MAMs play important roles in Ca^2+^ exchange, mitochondrial dynamics, and lipid metabolism. Numerous proteins in MAMs have been implicated in mitophagy (188). For example, Phosphofurin acidic cluster sorting protein-2 (PACS-2) plays a critical role in cellular functions by regulating the formation and transfer of cargo proteins at MAMs also in addition to regulating mitochondrial dynamics, endoplasmic reticulum stress, Ca^2+^ transport, autophagy, and apoptosis (189). Studies have shown that ox-LDL induces clustering of PACS-2 at mitochondria–endoplasmic reticulum contact sites, thereby increasing MAM contacts to promote the formation of phagosomal membranes (190, 191). The knockdown of PACS-2 interferes with mitochondrial formation and mitophagy, thereby increasing apoptosis of VSMCs, which may contribute to the formation of unstable AS plaques.

Recently, the disruption of lipid homeostasis in AS has received increased attention. Cardiolipin (CL) is a phospholipid molecule that exists in the IMM and translocates to the OMM upon mitochondrial damage. Externalized CL can interact with the dynamin-related protein Drp1 and promote its oligomerization, which is crucial for mitochondrial fission (192, 193). Furthermore, externalized CL induces a proinflammatory response. The proinflammatory response is associated with the activation of NLRP3 inflammasome (194, 195) and the activation of inflammatory signal transduction upstream from NF-κB (196). In addition, CL is particularly susceptible to lipid peroxidation. Oxidized CL increases the concentration of Ca^2+^ in monocytes/macrophages and neutrophils, and elevates the levels of the adhesion cytokines ICAM-1 and VCAM-1 in ECs (197). The hydrolysis of CL may lead to endothelial barrier dysfunction and necrosis (198). The accumulation of oxidized CL in OMM, leading to mPTP opening, and the release of cytochrome c from the mitochondria to the cytosol, induces apoptosis and programmed cell death (199, 200). Notably, externalized CL interacts with LC3 at the OMM, identifying damaged and dysfunctional mitochondria, and initiating mitophagy (201). Further research has found that CL accumulates at MAMs after mitophagy stimulation, interacting not only with MFN2 and CANX, but also with the multiple molecular complexes (AMBRA1/BECN1/WIPI1) involved in autophagosome formation (202). This suggests that CL plays an important role in both the early and late stages of mitophagy. Inhibition or knockdown of the convertase catalyzing the synthesis of CL with a high peroxidation index promotes mitophagy by up-regulating the expression of PINK1 and promoting the recruitment of Parkin to dysfunctional mitochondria, thereby alleviating oxidative stress, insulin resistance, and mitochondrial dysfunction (203–205). Although there no studies have yet shown a relationship between CL-related mitophagy and AS, it has been found that antibodies to oxidized CL correlate with the extent of AS progression, which warrants further investigation (206).

Ceramides are a class of bioactive lipids with structural and regulatory functions. Functionally, ceramide inhibits respiratory chain activity, induces ROS production and oxidative stress, disrupts ΔΨm, and may even induce apoptosis (207, 208). There is a large amount of evidence that ceramides are involved in the pathophysiological process of AS. Studies have shown that a high ceramide risk score is significantly associated with a higher risk of all-cause death in patients with coronary artery disease (209, 210). From the cellular level, ceramide may affect the activity of ECs by directly controlling NO levels (211, 212), promoting the transformation of macrophages into foam cells, mediating the apoptosis of foam cells (213), and inducing osteogenic differentiation of VSMCs (214). Recently, Vos M. et al. (215) showed that the accumulation of mitochondrial ceramide is sufficient to damage the mitochondrial electron transport chain, increase ROS production, and promote mitophagy. The ceramide in the mitochondrial membrane interacts directly with LC3-II on the autophagosomal membrane, thereby selectively targeting mitochondria for autophagy (216). Unfortunately, there has been no study on the relationship between ceramide and AS in the context of mitophagy.

## 3 Mechanisms and therapeutic advances of mitophagy in cells

### 3.1 ECs

#### 3.1.1 Mitophagy, ROS, and oxidative stress in ECs

Under normal conditions, ECs lining the inner surface of blood vessels maintain vascular homeostasis by regulating antithrombotic and anti-inflammatory processes, vascular tone, adhesion, and proliferation of VSMCs (217). In the development of AS, various factors cause endothelial dysfunction, resulting in vascular insufficiency, permeability, and proliferation of VSMCs, among other effects (218). However, a growing number of studies have shown that mitophagy plays an important role in endothelial dysfunction in AS. Mitochondria produce the vast majority of ROS, promoting poor activation/function of ECs, which leads to increased intravascular thrombosis, leakage, and inflammation (219–221). Therefore, regulating ROS-triggered oxidative damage in ECs may be an important way to prevent AS exacerbation.

Copper oxide nanoparticles can directly trigger oxidative stress and inflammation, leading to significant genotoxicity and cytotoxicity. Zhang et al. (222) have shown that copper oxide nanoparticles are ubiquitously deposited in lysosomes, leading to lysosomal dysfunction, impairment of autophagic flux, and accumulation of undegraded autophagosomes. They also induce EC death through a caspase-independent pathway, resulting in endothelial lesions. The mitophagy inhibitor Mdivi-1 specifically inhibits mitophagy, significantly exacerbating copper oxide nanoparticle-induced death of ECs (223). Activation of PINK1-mediated mitophagy contributes to the removal of damaged mitochondria in copper oxide nanoparticle-induced vascular ECs (224). This indicates that mitophagy has a protective effect on the copper oxide nanoparticle-induced death of ECs.

Some substances, such as melatonin and hydrogen sulfide, have been shown to exert a protective effect on ECs by alleviating oxidative stress damage through the regulation of mitophagy-related pathways. Tert-butyl hydroperoxide (TBHP) is a potent oxidizing agent that is commonly used to induce oxidative stress in ECs (225, 226). Oxidative stress is a key contributor to the development of AS. Melatonin reverses the damage in ECs caused by TBHP by enhancing the phosphorylation of AMPKα, promoting transcription factor EB (TFEB) nuclear translocation, up-regulating the protein levels of LC3, Parkin, and PINK1, and decreasing p62 (227). This indicates that melatonin may protect ECs against oxidative stress injury *via* Parkin and AMPK-TFEB mediated-mitophagy. In addition, hydrogen sulfide is a direct scavenger of ROS and peroxynitrite. It can protect cells from oxidative stress by reducing mitochondrial ROS production, increasing ATP synthesis, and preventing mitochondrial membrane depolarization at the cardiovascular and cellular levels (228). Cysteine β-synthase is the predominant hydrogen sulfide-producing enzyme in ECs. Silencing cysteine β-synthase in ECs significantly increases ROS production and ultimately reduces the lifespan and resilience of cells by affecting the mechanisms involved in MFN2 down-regulation, uncoupling of endoplasmic reticulum mitochondrial contact, increasing mitochondrial fission, and enhancing BNIP3-induced mitophagy (229). However, exogenous hydrogen sulfide may protect aortic ECs from high glucose and palmitate by inhibiting apoptosis and oxidative stress and up-regulating mitophagy through the PINK1/Parkin signaling pathway (230).

Notably, p66Shc acts as an oxidoreductase that can generate ROS by oxidizing cytochrome c. Piao et al. (231) have shown that the knockdown of p66shc inhibits mitochondrial ROS increase, membrane potential depolarization, and mitochondrial fusion triggered by the deletion of the CR6-interacting factor CRIF1. It also inhibits CRIF1 deficiency-induced mitophagy by reducing the levels of LC3-II/I, PINK1, and Parkin. Indeed, autophagy initiation requires processes such as autophagosome formation, fusion of autophagosomes with lysosomes, and degradation of autophagy substrates in lysosomes. LC3 is mainly involved in the formation of autophagosomes. In this study, only the expressions of LC3-II/I, PINK1, and Parkin, and the co-localization of LC3 with the nucleus, are observed, which cannot prove that the subsequent procedures of autophagy would take place.

#### 3.1.2 Mitophagy, glucose, and lipids in ECs

Exposure of ECs to high levels of glucose and lipids induces excessive production of ROS and reduces mitophagy, thereby accelerating the accumulation of dysfunctional mitochondria. This, in turn, results in poor EC function and apoptosis, and ultimately contribute to the development and progression of AS (232–235). Similarly, mitophagy deficiency in ECs accelerates the accumulation of dysfunctional mitochondria in ECs and triggers oxidative stress, thereby accelerating the aging of vascular ECs (236). Specifically, the most common type of mitophagy in ECs under high-glucose and -lipid conditions is PINK1-/Parkin-induced mitophagy. High levels of glucose and lipids decrease the expression of PINK1, Parkin, LC3II, Beclin1, and other mitophagy-related proteins in ECs, whereas the expression of p62 is up-regulated (8, 230, 236). Knockout of PINK1 or Parkin may inhibit mitophagy, resulting in the accumulation of mitochondrial fragments and leading to mitochondrial dysfunction, ROS overproduction, and apoptosis in ECs (237). Interestingly, the advanced glycation end products and their main precursor (methylglyoxal) may play an important role in the progression of AS and plaque rupture. Glycosylation leads to structural modifications and functional changes in the fibrinogen molecule, forming a cleavage-resistant, dense, and less porous fibrin network that leads to the progression of AS in diabetic patients (238, 239). The oxidative stress associated with the diabetic metabolite methylglyoxal may cause mitochondrial damage and the activation of Parkin-mediated mitophagy, resulting in the dysregulation of junction proteins and impaired permeabilization of ECs (240). Furthermore, Li et al. (233) have suggested that ox-LDL-induced endothelial injuries may be associated with PTEN overexpression. Inhibition of PTEN expression protects ECs by activating the AMPK/cAMP-response element-binding protein CREB/MFN2/mitophagy signaling pathway (233). These studies suggest that high glucose and lipid levels inhibit mitophagy in ECs by reducing the expression levels of ubiquitin-mediated mitophagy proteins.

Several drugs have shown properties that modulate mitophagy function (as shown in **Tables 1**, **2**). The combination of rivaroxaban and aspirin may increase the expressions of PINK1 and Parkin, restore ΔΨm, and decrease the level of ROS in high-glucose-induced ECs (263). Scutellarin, a plant extract, up-regulates mitophagy through the PINK1/Parkin signal pathway, thereby resisting hyperglycemia-induced endothelial injury (244). Moreover, resveratrol also reduces hyperlipidemia-induced endothelial injuries by enhancing BNIP3-associated mitophagy (241). This implies that promoting mitophagy protects mitochondrial integrity and prevents EC damage induced by high levels of glucose and lipids.

**Table 1:** Progress of natural compounds in regulating mitophagy in AS.

Molecule	Model	Treatment	Effects of mitophagy	Function	Reference
Resveratrol	HUVEC	ox-LDL	The levels of BNIP3, Atg5, Beclin1, and AMPK increased	It enhances mitophagy, reduces endothelial dysfunction, and protects ECs	(241)
13-Methylberberine	HUVEC	H_2_O_2_	Levels of LC3-II/I increased, and levels of p62 decreased	It enhances mitophagy, reduces endothelial dysfunction, and protects ECs	(242)
Puerarin	HUVEC	LPS	Levels of Atg5, Atg7, and Parkin increased	It enhances mitophagy, reduces endothelial dysfunction, and protects ECs	(243)
Scutellarin	HUVEC	High glucose	Levels of LC3-II, Beclin1, Atg5, PINK1, and Parkin increased, and levels of p62 decreased	It enhances mitophagy, reduces endothelial dysfunction, and protects ECs	(244)
GSCE	HAEC	High glucose and palmitic acid	Levels of LC3B-II, PINK1, Parkin, and p-AMPK increased, and levels of p62 decreased	It decreases mitophagy and protects ECs	(245)
Aloe-emodin derivative	HAEC, ApoE^-/^mice	HFD	Levels of LC3 II/I, AMBRA1, Beclin1, and Parkin increased, and levels of p62 decreased	It enhances mitophagy in ECs	(246)
Salvianolic acid B	HUVEC, db/db mice	High glucose or CCCP	Levels of Beclin1, Parkin, and PINK1 decreased	It enhances mitophagy, reduces endothelial dysfunction, and protects ECs	(247)
SL	CMECs	Oxygen–glucose deprivation	Levels of p62, LC3-II/I, PINK1, and Parkin decreased	It inhibits mitophagy and protects ECs function	(248)
ECE	VSMC	Serum from SHR	Levels of BNIP3, Atg12, and LC3-II/I increased, and levels of p62 decreased	It enhances mitophagy and decreases vascular calcification	(249)
Astaxanthin	VSMC	Ang II	Levels of PINK1 and Parkin increased	It enhances mitophagy and restrains the proliferation of VSMCs	(250)
Astragaloside IV	VSMC	Ang II	Levels of Parkin increased	It enhances mitophagy and protects cellular mitochondrial function	(251)
Celastrol	VSMC	Ang II	Levels of Parkin and PINK1 increased	It reduces oxidative stress and maintains mitochondrial homeostasis in VSMCs	(252)
Taurine	THP-1	LPS/IFN-γ	Levels of Parkin and p62 increased, and levels of PINK1, Beclin1, LC3-II/I, and VDAC1 decreased	It blocks mitophagy and reduces excessive proinflammatory polarization of macrophages	(253)
Fucoxanthin	RAW 264.7	Palmitic acid	Levels of p62, PINK1, Parkin, p-AMPK, BNIP3, Beclin1, and Atg5 increased	It enhances mitophagy, modulates lipid metabolism, and decreases inflammation	(254)
Artemisinin	RAW264.7, ApoE-/- mice	HFD, ox-LDL	Levels of LC3-II/I and p-AMPK increased, and levels of AMPK, p-ULK1, p-mTOR, and p62 decreased	It enhances autophagy and suppresses inflammatory responses	(255)
Alliin	THP-1	LPS	Levels of LC3-II, PINK1, and Parkin increased, and levels of p62 decreased	It enhances mitophagy and attenuates pyroptosis	(256)

CCCP, carbonyl cyanide 3-chlorophenylhydrazone; CMECs, cardiac microvascular endothelial cells; ECE, Ecklonia cava extract; GSCE, Ginseng Panax notoginseng Chuanxiong extract; HAEC, human aortic endothelial cell; IFN-γ, interferon γ; SHR, spontaneously hypertensive rat; SL, Shenlian extract.

**Table 2:** Progress of drug regulation of mitophagy in AS.

Molecule	Model	Treatment	Effects of mitophagy	Function	Reference
Melatonin	VSMC	β-GP	Levels of LC3-II/I, p-AMPK, p-ULK1, and Beclin1 increased, and levels of p62 decreased	It enhances mitophagy and inhibits VSMC calcification, apoptosis, and inflammation	(257)
	VSMC	β-GP	Levels of LC3-II/I and p-AMPK increased, and levels of p62 decreased	It enhances mitophagy, inhibits apoptosis, and attenuates the osteogenic-like phenotype transition of VSMCs	(258)
	HUVEC, flap model rat	TBHP	Levels of LC3B-II, PINK1, Parkin, and p-AMPK increased, and levels of p62 decreased	It activates mitophagy and alleviates oxidative stress and apoptosis	(227)
	RAW264.7, ApoE^–/–^ mice	ox-LDL, HFD	Levels of LC3-II/I and Parkin increased	It activates mitophagy and inhibits NLRP3 inflammasome activation	(259)
Pitavastatin	EPC, ApoE^–/–^ mice	HFD	Levels of LC3B-II, PINK1, and Parkin increased, and levels of p62 decreased	It enhances mitophagy, protects EPC proliferation, and promotes vascular re-endothelialization	(260)
Vitamin D	HUVEC	PM2.5 and high glucose	Levels of LC3B, BNIP3, and p62 decreased	It eliminates oxidative stress, inhibits mitophagy, and protects cells	(261)
Dexmedetomidine	RAW 264.7	LPS	Levels of LC3B-II and PINK1 increased	It enhances mitophagy and alleviates apoptosis and inflammation	(262)
Liraglutide	HUVEC	High glucose	Levels of LC3B-II, PINK1, and Parkin increased	It enhances mitophagy, reduces endothelial dysfunction, and protects ECs	(232)
Rivaroxaban and aspirin	HCAEC	High glucose	Levels of Parkin and PINK1 increased	It enhances mitophagy, inhibits ROS production, and protects ECs	(263)
Coenzyme Q10	HAEC and HUVEC	NRTI	Levels of LC3II/I decreased	It inhibits mitophagy and reduces endothelial dysfunction	(264)
	RAW264.7	LPS/ATP	Levels of LC3II/I, Parkin, Beclin1/Bcl-2, and p62 increased	It enhances mitophagy and inhibits NLRP3 inflammasome activation	(265)

β-GP, β-sodium glycerophosphate; EPC, endothelial progenitor cell; HAEC, human aortic endothelial cell; HCAEC, coronary artery endothelial cell; HFD, high-fat diet; NRTI, nucleoside reverse transcriptase inhibitor; TBHP, tert-butyl hydrogen peroxide.

In contrast, another study has found that ox-LDL can up-regulate the expression of NR4A1, a member of nuclear receptor subfamily 4A, and CaMKII, a calcium/calmodulin-dependent protein kinase, by post-translational modification, thereby activating Parkin-mediated mitophagy (266). The lack of NR4A1 mitophagy protects excess aortic ECs from ox-LDL-induced apoptosis. There are two possible reasons for this phenomenon. First, excessive mitophagy may consume mitochondrial mass, leading to an energy shortage and poor mitochondrial function. Second, acute lipid challenge up-regulates cellular autophagy in response to injury, but long-term lipid stimulation decreases cellular autophagy by inhibiting autophagosome–lysosome fusion (267). In chronic lipid loading, decreased macrolipophagy may promote cellular lipid accumulation, forming a vicious cycle of excessive lipid accumulation. In addition, human umbilical vein endothelial cells (HUVECs) exposed to particulate matter exhibit a significant increase in ROS production, inflammatory response, mitophagy, and apoptosis under high-glucose conditions (261). Vitamin D effectively attenuates oxidative stress, mitophagy, and inflammation in mice induced by streptozotocin and particulate matter (261). Moreover, in the thoracic aorta of diabetic mice with high-glucose- and carbonyl cyanide 3-chlorophenylhydrazone (CCCP)-induced ECs, salvianolic acid B significantly increases the expression of the apoptosis regulator Bcl-2, decreases the expression of the apoptosis regulators Bax, Beclin1, Parkin, and PINK1, inhibits mitophagy, and protects ECs from apoptosis (247). By comparing the findings of these studies, we speculate that acute exposure to hyperglycemia is an acute stress condition. Cells repair mitochondrial dysfunction by activating mitophagy. However, long-term exposure to hyperglycemia causes decreased glycolysis and massive generation of ROS, which inhibits ATP production and thus leads to suppressed mitophagy (268).

#### 3.1.2 Mitophagy and hypoxia in ECs

Recent studies have demonstrated that the imbalance between oxygen supply and demand in the arterial vessel wall is a key contributor to AS and plaque instability (269). Indeed, hypoxia can cause ROS overproduction and poor mitochondrial function, leading to enhanced local inflammatory responses and EC injury and death and ultimately promoting the formation and progression of AS plaques (270–273). The beneficial effects of mitophagy activation in protecting cells after hypoxia are controversial. Some scholars believe that mitophagy activation can reduce hypoxia-induced impairment of endothelial function by improving mitochondrial quality control, reducing ROS production, and increasing cell viability and proliferation (274–276). Conversely, Wang et al. (277, 278) believe that inhibiting hypoxia-induced autophagy/mitophagy can reduce the inflammatory response, injury, and apoptosis in ECs. Li et al. (248) have found that Shenlian extract may inhibit mitophagy through the PINK/Parkin pathway and regulate mitochondrial function to prevent hypoxia-induced EC dysfunction.

In fact, there may be multiple reasons for the activation of autophagy in ECs under hypoxia. Autophagy can be considered a cell protection/stress adaptation mechanism that ECs activate in response to energy deprivation (hypoxia, ischemia, nutrient deprivation, etc.) to protect themselves and maintain their own functions. From the perspective of energy metabolism, hypoxia is often accompanied by the consumption of nutrients. This activates AMPK, which phosphorylates and activates ULK1 to initiate autophagy (279). Regarding autophagy procedures, autophagy requires the formation of autophagosomes, the fusion of autophagosomes with lysosomes, and the degradation of autophagy substrates in lysosomes. Although some experiments have observed autophagosome formation and upstream regulatory mechanisms, this does not necessarily mean that mitophagy can successfully occur at the end or reverse the impairment due to insufficient mitophagy.

In the aforementioned studies, hypoxia resulted in the appearance of a large number of autophagosomes in tissues and cells, visible under transmission electron microscopy, and increases in the level of lysosomes co-localized with autophagosomes. However, the investigators concluded that hypoxia-induced hyperactivation of mitophagy, the opening of swollen mitochondria, fragmentation of mitochondrial ridges, and even fragmentation of the cytoplasm continued, although the ROS-scavenging ability of autophagosomes was reduced. (248). Existing evidence suggests that excessive accumulation of autophagosomes can interfere with cell function by damaging organelles and disrupting cell transport, ultimately leading to cell death (280). Moreover, under long-term stress conditions such as nutrient deprivation, hypoxia/ischemia, and oxidative damage, excessive activation of autophagy leads to excessive self-consumption and bioenergetic failure (281). Nevertheless, the detailed mechanism by which cell death occurs is unclear.

Collectively, mitophagy in ECs is induced by high blood glucose and lipid levels, ROS, hypoxia, and other vascular risk factors. Basal mitophagy is required to maintain EC function. However, insufficiently activated mitophagy to eliminate damaged mitochondria impairs vascular endothelial function, thereby promoting the development of AS. Under long-term stress conditions such as nutrient deprivation, hypoxia/ischemia, or oxidative stress, mitophagy may lead to cell death in a time-dependent manner.

### 3.2 VSMCs

#### 3.2.1 Mitophagy and ox-LDL in VSMCs

VSMCs are a significant cellular component of the blood vessel wall, which regulates blood pressure and blood flow through vasoconstriction and vasodilation (282). Under physiological conditions, differentiated VSMCs are located in the arterial intima and have a contractile phenotype. It is characterized by less proliferation, less extracellular matrix secretion, and the expression of specific contractile proteins such as smooth muscle myosin heavy chain, smooth muscle α-actin, and calmodulin (283–285). However, vascular damage results in the loss of VSMC contractility and a transition to a proliferative phenotype, migrating to the intimal layer of the vessel wall, which accelerates the formation of AS plaques (286). With the development of AS plaques, the apoptosis and extracellular matrix degradation of VSMCs increase, rendering them more susceptible to rupture (287). Therefore, the regulation of VSMC phenotype, proliferation, and death is the key to stabilizing AS plaques.

In VSMCs, ox-LDL may induce mitochondrial DNA damage in AS plaques, leading to decreased aerobic respiration in the cap and core regions (288) and promoting VSMC phenotype conversion (289) and calcification (290), ultimately resulting in VSMC apoptosis (291). Apoptosis of VSMCs in the plaque promotes the formation of unstable plaques (292). Recent studies have shown that mitophagy regulates the VSMC phenotype and proliferation, and can prevent apoptosis caused by low levels of ox-LDL (293) or autophagy (294). First discovered by Swiader et al. (293), knockdown of PINK1 or Parkin impairs mitophagy flux and increases ox-LDL-induced apoptosis in VSMCs. However, overexpression of PINK1 and Parkin can activate granulocyte autophagy and protect VSMCs. It may also suppress NLRP3 inflammasome hyperactivation to reduce inflammation in AS plaques (295) and promote a compensatory glycolytic program linked to AMPK and Hex2 in response to mitochondrial dysfunction (296).

#### 3.2.2 Mitophagy, ROS, and oxidative stress in VSMCs

The production of ROS and oxidative stress are important factors contributing to VSMC proliferation. Several studies have demonstrated the role of ROS in VSMC proliferation. For example, hydrogen peroxide (H_2_O_2_), a type of ROS, was found to induce the proliferation of VSMCs *in vitro* (297, 298). Similarly, angiotensin II (Ang II) is a peptide hormone that regulates blood pressure and fluid balance. Studies have shown that it can cause mitochondrial fragmentation and dysfunction, leading to increased production of ROS and oxidative stress in VSMCs (250). This can promote the proliferation and migration of VSMCs (297, 299). Geng et al. (252) demonstrated that nuclear receptor NR4A1 controls mitophagy and mitochondrial fission in VSMCs to maintain mitochondrial homeostasis and protect VSMCs from Ang II-induced oxidative stress damage. Celastrol restores mitochondrial autophagy flux in VSMCs through up-regulating nuclear receptor NR4A1 expression. Meanwhile, Wang et al. (300) suggested that corticostatin can inhibit mitophagy through its receptor somatostatin receptor types 3 and 5, thereby reducing the Ang II-induced production of ROS and proliferation of VSMCs.

Vascular calcification is a marker of vascular stiffness in AS. It is caused by advanced glycation end products and excess ROS (301–303). Excessive ROS production can lead to oxidative stress, which has a destructive effect on lysosomal membrane integrity, resulting in the release of lysosomal hydrolases and insufficient mitophagy after prolonged exposure (301, 304, 305). This is primarily connected to the onset and development of vascular calcification. The change in VSMC phenotype is a crucial factor in vascular calcification, and the transition from a contractile to an osteogenesis phenotype promotes vascular calcification (306). There have been groundbreaking advances in the regulation of VSMC phenotype conversion with certain drugs. Melatonin protects VSMCs from calcification by promoting mitochondrial fusion and mitophagy through the AMPK/mTOR/ULK1 pathway, thus reducing the risk of AS (307). In this process, the dwarf-related protein Runx2 and cleaved caspase-3, which regulate osteogenic phenotype and transcription factors, are down-regulated. This indicates that mitophagy and mitochondrial fusion play vital roles in regulating the phenotype of VSMCs and the development of vascular calcification, which is a breakthrough in reducing the risk of AS. In addition, metformin has also been proven to affect mitochondrial biogenesis by increasing mitophagy, thereby preventing β-glycerophosphate-induced osteogenic phenotypic transformation of VSMCs (258).

Aging is a well-known risk factor for AS. Oxidative stress and ROS may be key to the link between aging and vascular calcification (308). Tyrrell et al. (309, 310) have shown that aging in the presence of normal lipids can lead to mitochondrial dysfunction, the activation of mitophagy, and an increase in the levels of the proinflammatory cytokine IL-6 in the aorta. Meanwhile, enhancing mitophagy by adding spermidine to drinking water during hyperlipidemia inhibits aortic mitochondrial dysfunction, IL-6 levels, and the development of AS with age (311). Excessive accumulation of lipids is an important factor that leads to vascular aging (312). Uchikado et al. (313) have found that ox-LDL induces mitochondrial division *via* the combination of lectin-like ox-LDL scavenger receptors and angiotensin type II1 receptors, and the rapidly accelerated fibrosarcom (RAF) proto-oncogene serine/threonine-protein kinase CRAF/mitogen-activated protein kinase MEK/extracellular signal-regulated kinase ERK pathway, causes massive production of ROS, leading to mitochondrial dysfunction and aging. However, inhibition of the angiotensin type II1 receptor attenuates senescence by blocking this cascade and inducing the replacement of autophagy by mitophagy through the CRAF/MEK axis and mitochondrial quality control processes in human VSMCs, which are dependent on Ras-related protein (313). Additionally, astragaloside IV alleviates the structural and biochemical abnormalities caused by Ang II in VSMCs by eliminating mtROS levels and enhancing both mitophagy and mitochondrial biosynthesis (251).

#### 3.2.3 Mitophagy and metabolic reprogramming of VSMCs

Recent studies have shown that increased expression of pyruvate dehydrogenase kinase isotype PDK4 is associated with calcified vessels in patients with AS (314). Pyruvate produced during glycolysis is transported to the mitochondria under normoxic conditions, where it is converted to acetyl-CoA by pyruvate dehydrogenase complexes and then enters the tricarboxylic acid cycle (315, 316). However, under hypoxic conditions, pyruvate dehydrogenase complexes are inhibited, leading to reduced mitochondrial tricarboxylic acid cycling activity and increased conversion of pyruvate to lactate in the cytoplasm, known as the Warburg effect (317, 318). This metabolic shift is observed in calcified vessels in patients with AS (314). Recent research has shown that the metabolic reprogramming of VSMCs toward high-rate glycolysis and lactate production in the cytosol is driven by PDK4 (319). However, PDK4 inhibition promotes lysosomal activity and mitophagy, reducing VSMC calcification, and alleviating AS (319). In addition, lactic acid inhibits BNIP3-mediated mitophagy, leading to VSMC calcification *in vitro* (320). Further research has revealed that lactate may inhibit mitophagy by inducing apoptosis and accelerating the phenotypic transition of osteoblasts and calcium deposition in VSMCs through activation of the NR4A1/DNA-dependent protein kinase catalytic subunit/cell tumor antigen p53 pathway (321). Notably, metabolic reprogramming and mitophagy seem to interact (322). Mitophagy controls metabolic reprogramming, which has an impact on how cells develop and differentiate (323, 324). Therefore, targeting mitophagy and metabolic reprogramming in VSMCs may hold promise as novel treatments for AS.

#### 3.2.4 Mitophagy in VSMCs may not always be beneficial

The migration of VSMCs from the vascular media to the intima is a key factor in the pathogenesis of AS. Microtubules, an important part of the cytoskeleton, play an important role in VSMCs’ migration. A balance between dynamically unstable and stable microtubules is required for cell migration, and the microtubule stability is regulated by multiple microtubule-associated proteins and post-translational modifications (325). KAT2a is a histone acetyltransferase that increases the acetylation of tubulin-α (326). Acetylation of tubulin-α increases the polymerization and stability of microtubules, ultimately inhibiting the directional migration of cells (327, 328). However, activation of autophagy selectively degrades KAT2a, disrupts the microtubule stability, and promotes the directional migration of VSMCs (329). Further research has revealed that specific deletion of ULK1 in VSMCs can inhibit the autophagic degradation of KAT2A, which increases the protein level of acetylated tubulin-α and inhibits the directional migration of VSMCs and the formation of new intima (330). Thus, inhibiting autophagy in VSMCs may be a crucial therapeutic strategy for AS. The relationship between autophagy and microtubule stability remains controversial. Choi et al. (331) have found that glucocorticoids induce microtubule dysfunction and inhibit autophagy by activating mTOR, increasing the protein level of SCG10, a microtubule destabilizing protein, and finally inducing microtubule instability. Autophagy increases microtubule stability by degrading SCG10 and promotes axonal regeneration after injury (332). Kirchenwitz et al. (333) have found that SMER28, an inducer of autophagy, significantly alters microtubule dynamics in cells, promotes acetylation of microtubules, and increases resistance to excitotoxin-induced axonal degeneration.

Apelin is an endogenous ligand of the G protein-coupled receptor APJ. Both apelin and APJ receptors are expressed in VSMCs and exert a variety of effects on the cardiovascular system, namely, angiogenesis, vasodilation, and cardiac contractility (334). He et al. (335) have shown that in the AS lesions of ApoE^-/-^ mice and VSMCs induced by apelin-13, the expression of several mitophagy-related proteins, such as PINK1, Parkin, p-AMPKα, and VADC1, is significantly up-regulated. They believe that PINK1/Parkin-mediated mitophagy promotes apelin-13-induced human aortic VSMC proliferation by activating p-AMPKα and exacerbates the progression of AS lesions *in vivo*. Furthermore, Chen et al. (336) have confirmed this view further and found that mitochondrial calcium uniporter uptake-dependent mitochondrial calcium-induced mitophagy is involved in the proliferation of VSMCs induced by apelin-13. In fact, some scholars believe that apelin and APJ receptors are protective against AS. Kostopoulos et al. (337) have found that apelin expression is negatively correlated with AS by analyzing human aorta and coronary arteries using immunohistochemical staining, concluding that apelin and its APJ receptor have anti-AS effects in human arteries. Chun et al. (338) have found that apelin produces an anti-AS effect by promoting nitric oxide production and inhibiting the cell signaling of Ang II. Additionally, apelin-13 treatment improves blood lipid levels and reduces the vulnerability of AS plaque (339). Apelin inhibits the increased proliferation of cells and blocks the progression of the cell cycle in VSMCs in response to hypoxia. This may be due to apelin's inhibition of autophagy levels in VSMCs. Zhang et al. (340) have shown that apelin protects VSMCs from apoptosis and inhibits the migration of VSMCs under hypoxic conditions. Another study found that apelin can inhibit the osteogenic differentiation of VSMCs and has a protective effect on the calcification of arteries (341). Therefore, the role of the apelin/APJ system in VSMCs needs further investigation.

In summary, most scholars believe that autophagy deficiency in VSMCs promotes an unstable plaque phenotype in AS (342). As a regulatory mechanism of mitochondrial homeostasis, mitophagy may protect VSMCs by regulating ROS generation, oxidative stress, and metabolic levels and by preventing VSMCs from phenotype change, proliferation, and death. This provides possibilities for the prevention and treatment of atherosclerotic, unstable plaques. Although current studies believe that inhibition of autophagy can prevent abnormal proliferation and migration of VSMCs, the degree and duration of autophagy are essential for cell health, and different stress conditions may also be significant reasons for the divergence of research conclusions. In conclusion, new therapies for AS that focus on mitophagy in VSMCs may show great prospects and need further investigation.

### 3.3 Macrophages

#### 3.3.1 Mitophagy and lipids in macrophages

Macrophages have the ability to actively engulf ox-LDL, leading to the expression of high levels of proinflammatory cytokines, such as TNF, IL-6, and IL-1β, and releasing matrix metalloproteinases during the development of AS (6). This weakens the stability of vulnerable AS plaques by hydrolyzing the collagen fibers in the fibrous cap. However, excessive lipid accumulation impairs mitophagy, which may be associated with excessive ROS generation and exacerbated inflammatory responses (343). Ultimately, this leads to macrophage dysfunction, secondary necrosis, and increased inflammation, which can manifest AS plaque erosion or rupture of vulnerable AS plaques. Mice deficient in FUNDC1 and stimulated by a high-fat diet show increased macrophage infiltration, enhanced M1 macrophage polarization, impaired mitophagy, and develop more severe obesity and insulin resistance (344). Onat et al. (345) have shown that lipid-activated eukaryotic initiation factor EIF2α signaling blocks Parkin-mediated mitophagy in macrophages, leading to greater mitochondrial oxidative stress, inflammasome activation, and IL-1β secretion. Gupta et al. (346) have shown that palmitate induces acetylation of the forkhead box protein FOXO3a, which inhibits the LPS-induced binding of FOXO3a to the PINK1 promoter. This inhibition may be related to LPS-induced mtROS production, mtDNA release, apoptosis-associated speck-like protein (ASC) oligomerization, and caspase-1 activation of macrophages under palmitate conditions. As a result, the activation of the NLRP3 inflammasome increases while PINK1-mediated mitophagy is reduced. The up-regulation of autophagy not only reduces the accumulation of intracellular lipid droplets but also inhibits cell apoptosis and the progression of AS by removing dysfunctional mitochondria and reducing intracellular ROS levels (347). Fucoxanthin treatment increases the expression of PINK1, Parkin, BNIP3, and p-AMPK and significantly increases p62 accumulation, and attenuates inflammation in raw 264.7 induced by palmitic acid (254). Notably, in addition to the high-fat diet, abrupt high-protein diet intake increases amino acid levels in the blood and AS plaques, promotes macrophage mTOR signaling to suppress mitophagy, exacerbates the build-up of dysfunctional mitochondria, and induces AS lipid-induced macrophage apoptosis (170).

#### 3.3.2 Mitophagy and NLRP3 inflammasome activation in macrophages

NLRP3 inflammasome activation and mitophagy must coexist in a balanced manner to maintain cellular and mitochondrial homeostasis, but the exact nature of this relationship is still unknown. Following oligomerization, NLRP3 binds to ASC, which contains the C-terminal caspase-recruitment domain, through its pyrin domain (348). The pro-cysteinyl aspartate-specific proteinase pro-caspase-1 is then recruited to the C-terminal caspase recruitment domain on ASC, resulting in the autocatalytic cleavage of pro-caspase-1 to its active form, caspase-1 (349, 350). However, inhibiting caspase-1 can increase mitophagy, exocytosis, and M2 polarization of macrophages, prevent foam cell formation, and inhibit NLRP3 inflammasome assembly, thereby reducing vascular inflammation and AS (351).

NF-κB is a well-known key activator of inflammation that induces NLRP3 inflammasome activation by up-regulating pro-IL-1β and NLRP3 expression (352–354). Zhong et al. (355) have found that NF-κB can prevent excessive inflammation and inhibit NLRP3 inflammasome activation by inducing delayed accumulation of autophagy receptor p62 in macrophages. It is beneficial for self-limited host response to tissue repair. This suggests that NF-κB has both proinflammatory and anti-inflammatory effects in macrophages, but the complex mechanism remains to be elucidated.

Endogenous lipidoids can activate the NLRP3 inflammasome, potentially driving metabolic inflammation relevant to the pathogenesis of AS (356). Ma et al. (259) have shown that melatonin can reduce the production of mitochondrial ROS by activating the silent information regulator sir2-like protein 3/FOXO3/Parkin-mediated mitophagy, thereby weakening the inflammatory activation of NLRP3 in macrophages induced by ox-LDL. In contrast, dietary polyunsaturated fatty acids may slow the development of AS by triggering macrophage autophagy and reducing NLRP3 inflammasome activation, reducing the number of dysfunctional mitochondria (357).

Recent research has demonstrated that the relationship between macrophages and heme is crucial to homeostasis and inflammation (358). Btb-and-cnc homolog 1 (BACH1) is a key regulator of the cell cycle, cell differentiation, oxidative stress response, and heme homeostasis, which are closely related to the occurrence and development of AS (359, 360). Pradhan et al. (361) have found that mitochondrial energy metabolism in BACH1^–/–^ macrophages shift toward increased glycolysis and reduced oxidative phosphorylation, resulting in increased ΔΨm and mitochondrial ROS production and reduced mitophagy. These changes ultimately trigger the activation of the NLRP3 inflammasome. This enhances the correlation between metabolic reprogramming and mitophagy.

Overall, understanding the relationship between the NLRP3 inflammasome and mitophagy is essential for maintaining macrophage and mitochondrial homeostasis and preventing the development of AS. Further research is needed to fully elucidate the complex mechanisms involved in these processes.

#### 3.3.3 Macrophage mitophagy and macrophage polarization

It is well known that several phenotypically different macrophages play different roles. M2 macrophages are associated with anti-inflammatory effects, tissue repair, and wound healing, whereas M1 macrophages are linked to proinflammatory effects and plaque rupture (362). The polarization classification of macrophages affects the progression and regression of AS. Choi et al. (343) have shown that apolipoprotein A-I binding protein (AIBP) is a novel regulator of macrophage autophagy. In AS, mitochondrial AIBP is involved in mitophagy and mitochondrial quality control, which reduces ROS production and prevents cell death (343). Duan et al. (363) suggest that mitochondrial AIBP may play an anti-AS role by regulating PINK1-dependent mitophagy and M1/M2 polarization.

Notably, reprogramming of macrophages from M1 to M2 can be achieved by targeting metabolic events. Taurine, one of the byproducts of S-adenosylmethionine’s metabolism downstream to the sulfur pathway, helps to maintain the homeostasis of the anti-inflammatory process and cellular energy metabolism (364). Meng et al. (253) have discovered that taurine inhibits methylation of the S-adenosylmethionine-dependent protein phosphatase 2 catalytic subunit, which blocks PINK1-mediated mitophagy flux. This maintains high mitochondrial density and eventually prevents the glycolytic conversion of energy metabolism to M1.

In addition, the cytokines IL-25 (365, 366) and IL-33 (367–369) are considered to have the potential to regulate the progression of AS. Lin et al. (370) have found that IL-33 stimulates the generation of ROS production and subsequently up-regulates the expression of PINK1, Parkin, and LC3 through the AMPK signaling pathway. Furthermore, IL-33 significantly decreases the production of the M1 macrophage-related cytokines CXCL-10 and TNF-α, while increasing the production of the M2 macrophage-related cytokine CCL-22. Tsai et al. (371) have demonstrated that IL-25 induces ROS production and significantly activates the expression of p-AMPK and PINK1, p-Parkin, and LC3 in a dose-dependent manner, promoting mitophagy and M2 macrophage polarization in THP-1-derived macrophages.

#### 3.3.4 Mitophagy and macrophage aging

Aging is an important factor affecting mitophagy in macrophages. The simulator of interference response protein STING is considered a cell solid DNA sensor that mediates sterile inflammation closely associated with atherosclerosis and aging (372, 373). Zhong et al. reported a decrease in PINK1/Parkin-mediated mitochondrial polyubiquitination in aging macrophages (370). In addition, aging changes caused impairment of lysosomal biogenesis and function in macrophages *via* regulation of the mTOR/TFEB signaling pathway. Overexpression of PINK1 did not promote mitochondrial lysosome formation in aged macrophages, but it did reverse the inhibitory effect of mitochondrial ubiquitination. Combining PINK1 overexpression and treatment with the mTOR inhibitor Torin-1 restored mitophagy flux in aged macrophages and attenuated STING activation. In addition, the guanine nucleotide-binding protein (Gbp1) plays an important role in regulating macrophage polarization, metabolic reprogramming, and cellular senescence (374). Qiu et al. (375) have shown that Gbp1 participates in the removal of damaged or unhealthy mitochondria brought on by proinflammatory cytokine stimulation during mitophagy. Specifically, Gbp1 plays a protective role against inflammation-induced macrophage inflammatory response and metabolic dysfunction by maintaining mitochondrial function and preventing mitochondrial dysfunction-associated senescence through the promotion of mitophagy. However, down-regulation of Gbp1 reduces mitophagy activity, leading to mitochondrial dysfunction, oxidative stress, inflammation, and aging.

In conclusion, dysregulation of lipid metabolism, inflammation, and aging are important factors leading to impaired mitophagy in macrophages. Mitophagy can prevent the formation and development of AS by removing dysfunctional mitochondria, reducing intracellular ROS levels, inhibiting NLRP3 activation, and regulating macrophage energy metabolism, among other pathways.

## 4 Conclusions

Recent research has demonstrated that the occurrence, development, and pathological mechanisms of AS are all closely related to mitophagy. Mitophagy dysfunction has been linked to several factors, such as ROS, glucose and lipid metabolism disorders, and hypoxia. This dysfunction may cause damage to ECs and result in the proliferation and phenotypic switching of VSMCs. Additionally, it can induce altered polarization of macrophages and metabolic dysfunction, and potentially lead to cell death. However, there are still many limitations and problems in the current research. There is a dearth of reports on animal or clinical studies, with most studies being limited to the cellular level. The correlation between these studies and AS requires additional verification. Is there a common or specific mitophagy pathway present in different cells or tissues within the study model? Does mitophagy in the same cells and tissues have different feedback in different stress modes, to varying degrees and over time? Can some stress conditions closely relate to AS, such as lipid accumulation, abnormal glucose metabolism, inflammation, and oxidative stress, only activate specific mitophagy pathways? It should be noted that further investigations are necessary to determine whether mitophagy is the preferred stress or compensatory pathway of cells under these stress conditions.

In terms of research mechanisms, the classical PINK1/Parkin pathway is currently receiving more attention, while research on other pathways is relatively scarce. In addition, there is a crosstalk between different mitochondrial autophagy pathways and classical AS mechanisms. The same protein can participate in various mitophagy pathways and respond to various stresses showing different expression patterns. It is worth noting that mitophagy can protect cells, but it may also lead to cell damage and cell death. This is an important issue that needs to be clarified in the future. In drug research, a variety of natural and auxiliary drugs that can directly or indirectly regulate mitochondrial quality control have been further explored. However, there is still a lack of safe and effective mitochondrial-targeted therapies. In conclusion, mitophagy, as an important mechanism for regulating AS, needs to be further explored.

## Author contributions

YZ and JW conceived the article and wrote the original draft. LH gathered materials and revised the manuscript. SS designed and drew pictures of the mechanism and reviewed the manuscript. FX reviewed the manuscript and critically revised important intellectual content. All authors contributed to the article and approved the submitted version.

## Glossary

AIBP: A-I binding protein
Ang II: angiotensin II
AMBRA1: activating molecule in Beclin 1-regulated autophagy protein 1
AMPK: adenosine 5′-monophosphate-activated protein kinase
AS: atherosclerosis
ASC: apoptosis-associated speck-like protein
Atg: autophagy-related protein
ATP: adenosine triphosphate
BACH1: Btb-And-Cnc homolog 1
BCL2L1: Bcl-2-like protein 1
BCL2L13: Bcl-2-like protein 13
BNIP3: BCL2-interacting protein 3
BNIP3L/Nix: BCL2 interacting protein 3-like
CANX: calnexin
CCCP: carbonyl cyanide 3- chlorophenylhydrazone
CK2: casein kinase 2
CL: cardiolipin
COMP: cartilage oligomeric matrix protein
DFCP1: double FYVE-containing protein 1
DRP1: dynamic-related protein 1
EC: vascular endothelial cell
EPC: endothelial progenitor cell
ER: endoplasmic reticulum
RB1CC1: RB1-inducible coiled-coil protein 1
FUNDC1: FUN14 domain-containing protein 1
H_2_O_2_: hydrogen peroxide
HAEC: human aortic endothelial cell
HFD: high-fat diet
HIF: hypoxia-inducible factor
HUVEC: human umbilical vein endothelial cell
Hcy: hyperhomocysteine
IL: interleukin
IMM: inner mitochondrial membrane
LC3: microtubule-associated protein 1 light chain 3
LIR: LC3- interaction region
LPS: lipopolysaccharide
MAM: mitochondria-associated endoplasmic reticulum
MARCH5: mitochondrial E3 ubiquitin ligase MARCH5
MFN1: mitofusin 1
MFN2: mitofusin 2
MPP: mitochondrial processing peptidase
mPTP: mitochondrial permeability transition pore
mTORC1: mTOR complex 1
NBR1: next to BRCA1 gene 1 protein
NDP52: nuclear domain 10 protein 52
NF-κB: nuclear factor-kappa B
NLRP3: NOD-like receptor thermoprotein dome-associated protein 3
OMM: outer mitochondrial membrane
NOD: nucleotide oligomerization domain
OPTN: optineurin
ox-LDL: oxidized low-density lipoprotein
PACS-2: phosphofurin acidic cluster sorting protein 2
PARL: presenilin associated rhomboid-like protein
PE: phosphatidyl ethanolamine
PGAM5: PGAM family member 5
PHB2: prohibitin 2
PI3P: phosphatidylinositol 3-phosphate
PINK1: PTEN-induced putative kinase protein 1
PTEN: phosphatase and tensin homolog
PTEN-L: phosphatase and tensin homolog-long
pUB: phosphorylated UB
Rab: Ras-related protein
ROS: reactive oxygen species
SQSTM1/p62: sequestosome-1/ubiquitin-binding protein p62
SRC: SRC proto-oncogene, non-receptor tyrosine kinase
TBHP: tert-butyl hydroperoxide
TBK1: tank-binding kinase 1
TIMM23: inner mitochondrial membrane 23 translocase
THP-1: human myeloid leukemia-derived
TNF: tumor necrosis factor
TOMM: translocase of the outer mitochondrial membrane
UB: ubiquitin
ULK1: unc-51-like autophagy activating kinase 1
UPS: ubiquitin-proteasome system
USP: ubiquitin-specific protease
VDAC1: voltage-dependent anion-selective channel protein 1
VEGFR2: vascular endothelial growth factor receptor 2
Vps: vacuolar protein sorting-associated protein
VSMC: vascular smooth muscle cell
WIPI2: WD repeat domain phosphoinositide-interacting protein 2
ΔΨm: mitochondrial membrane potential.
